# Cathepsin B Dipeptidyl Carboxypeptidase and Endopeptidase Activities Demonstrated across a Broad pH Range

**DOI:** 10.1021/acs.biochem.2c00358

**Published:** 2022-08-18

**Authors:** Michael C. Yoon, Vivian Hook, Anthony J. O’Donoghue

**Affiliations:** † Skaggs School of Pharmacy and Pharmaceutical Sciences, University of California, San Diego, La Jolla, California 92093, United States; ‡ Biomedical Sciences Graduate Program, University of California, San Diego, La Jolla, California 92093, United States; § Department of Neurosciences and Department of Pharmacology, School of Medicine, University of California, San Diego, La Jolla, California 92093, United States

## Abstract

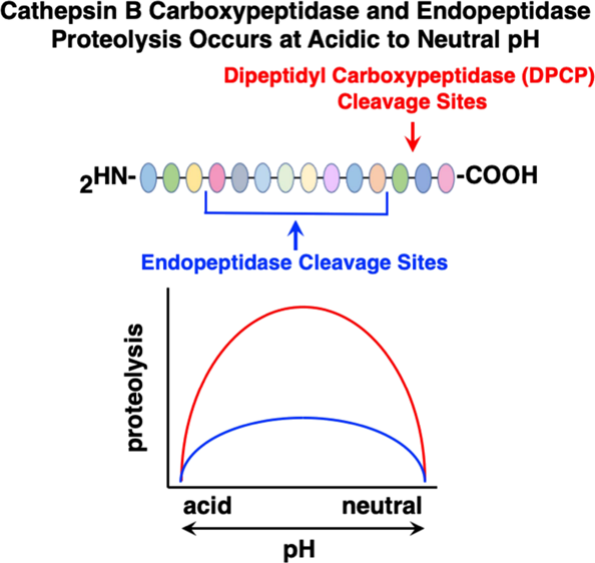

Cathepsin B is a lysosomal protease that participates in protein degradation. However, cathepsin B is also active under neutral pH conditions of the cytosol, nuclei, and extracellular locations. The dipeptidyl carboxypeptidase (DPCP) activity of cathepsin B, assayed with the Abz-GIVR↓AK(Dnp)-OH substrate, has been reported to display an acidic pH optimum. In contrast, the endopeptidase activity, monitored with Z-RR-↓AMC, has a neutral pH optimum. These observations raise the question of whether other substrates can demonstrate cathepsin B DPCP activity at neutral pH and endopeptidase activity at acidic pH. To address this question, global cleavage profiling of cathepsin B with a diverse peptide library was conducted under acidic and neutral pH conditions. Results revealed that cathepsin B has (1) major DPCP activity and modest endopeptidase activity under both acidic and neutral pH conditions and (2) distinct pH-dependent amino acid preferences adjacent to cleavage sites for both DPCP and endopeptidase activities. The pH-dependent cleavage preferences were utilized to design a new Abz-GnVR↓AK(Dnp)-OH DPCP substrate, with norleucine (n) at the P3 position, having improved DPCP activity of cathepsin B at neutral pH compared to the original Abz-GIVR↓AK(Dnp)-OH substrate. The new Z-VR-AMC and Z-ER-AMC substrates displayed improved endopeptidase activity at acidic pH compared to the original Z-RR-AMC. These findings illustrate the new concept that cathepsin B possesses DPCP and endopeptidase activities at both acidic and neutral pH values. These results advance understanding of the pH-dependent cleavage properties of the dual DPCP and endopeptidase activities of cathepsin B that function under different cellular pH conditions.

## Introduction

Cathepsin B is a member of the cysteine cathepsin family of lysosomal proteases^1^ participating in protein degradation and homeostasis.^2,3^ Recent studies indicate that the lysosome functions as a signaling complex for regulation of cellular functions in health and in disease.^4,5^ Cathepsin B is also located outside of acidic lysosomes under neutral pH conditions of the cytosol, nuclei, and extracellular locations in numerous human diseases including neurodegenerative diseases,^6−8^ cancer,^9−12^ autoinflammation,^13−15^ viral infection,^16^ and pathogen-induced pyroptosis.^17^ The translocation of lysosomal cathepsin B to the cytosol participates in lysosomal signaling regulation of cellular functions including inflammation and cell death in disease.^8^

Cathepsin B is active at the neutral pH 7.2^18,19^ of the cytosol and other neutral pH compartments of biological systems,^20,21^ as well as at the acidic pH 4.6 of lysosomes.^22,23^ Importantly, cathepsin B is primarily a dipeptidyl carboxypeptidase (DPCP) type of exopeptidase^18^ and also functions as an endopeptidase shown by cleavage of peptide and protein substrates at sites distal from the C-terminus of peptide substrates.^18,24,25^

Examination of DPCP activity of cathepsin B with internally quenched fluorogenic peptide substrates containing a free carboxy termini led to identification of 2-aminobenzoyl-Gly-Ile-Val-Arg-Ala-Lys(2,4-dinitrophenyl)-OH (Abz-GIVRAK(Dnp)-OH) as an excellent substrate for DPCP activity where cleavage occurs between R↓A to result in the dipeptide AK(Dnp)-OH product.^26^ This substrate is routinely used in the field for monitoring DPCP activity of cathepsin B.^1,27−29^ Evaluation of this substrate over a pH range of pH 2.5–8 showed optimal DPCP activity at acidic pH 4.5.^26^ In contrast, cathepsin B activity with Z-Arg-Arg-7-amino-4-methylcoumarin (Z-RR-AMC), an endopeptidase substrate, displayed highest cleaving activity at neutral pH.^30−32^ Peptide-AMC substrates with a blocked N-terminus (e.g., Z-group) have been extensively used to quantify endopeptidase activity^33,34^ and, therefore, it has been assumed that Z-RR-AMC is also detecting endopeptidase activity of cathepsin B. From this interpretation, it has been widely thought that the DPCP activity of cathepsin B decreases as pH increases,^31,35,36^ while endopeptidase activity increases as pH increases.^29,37−40^

Previous studies by our group have shown that peptide cleavage efficiencies by cathepsin B under different pH conditions are dependent on the sequences of the peptide substrates.^18^ We, therefore, questioned whether the pH-dependent differences that have been observed for Abz-GIVRAK(Dnp)-OH and Z-RR-AMC cleavages by cathepsin B may be due to differences in substrate sequences rather than a pH-dependent transition between DPCP and endopeptidase activities. To address this question, we evaluated the DPCP and endopeptidase cleavage profiles of cathepsin B under acidic and neutral pH conditions with a peptide library using the approach of multiplex substrate profiling by mass spectrometry (MSP-MS).^18,41,42^ The library consists of 228 14-mer peptides, and cleavage at any one of the 2964 peptide bonds can be determined by LC–MS/MS.^43^ The cleavage profiling of cathepsin B was performed in parallel with the endopeptidase cathepsin K to demonstrate the difference between DPCP and endopeptidase activities. Cathepsin K was selected for these control studies because it is a cysteine protease homologous to cathepsin B, both enzymes are active at pH 4.6 and pH 7.2, both cleave the substrate Z-FR-AMC,^18,44^ yet cathepsin B has dual DPCP and endopeptidase activity while cathepsin K has only endopeptidase activity.^45,46^

Data from this study illustrate the new concept that cathepsin B displays both DPCP and endopeptidase activities under acidic to neutral pH conditions, illustrated by novel substrates designed based on cleavage properties under different pH conditions. These results change the widely held view that cathepsin B transitions from having exopeptidase to endopeptidase activity as the pH increases^31,35−39^ to the new finding that both types of activities occur under acidic to neutral pH conditions. To illustrate this new concept of cathepsin B cleavage properties, novel peptide substrates were designed based on pH-dependent cleavage profiling features of this enzyme. We showed that the new DPCP substrate Abz-GnVRAK(Dnp)-OH is cleaved efficiently across a broad pH range, while the endopeptidase substrate Z-ER-AMC was cleaved more efficiently at acidic pH compared to Z-RR-AMC. This study demonstrates the new concept that the major DPCP and minor endopeptidase activities of cathepsin B both occur under acidic and neutral pH conditions.

## Methods and Procedures

### Cathepsin B Fluorogenic Activity Assays

Recombinant human cathepsin B was purchased from R&D Systems (Minneapolis, MN) and was activated to mature cathepsin B by incubation at 37 °C for 30 min in activation buffer (20 mM Na-acetate pH 5.5, 1 mM EDTA, 5 mM DTT, 100 mM NaCl). For the pH profiling, cathepsin B activity was monitored over the pH range of pH 2.2–9.0 in 40 mM citrate phosphate (pH 2.2 to pH 7.4) or 40 mM Tris–HCl (pH 7.8–9.0), 1 mM EDTA, 100 mM NaCl, and 5 mM DTT, with preincubation in each pH buffer for 10 min prior to initiating the assay by adding 40 μM substrate. The substrates Z-VR-AMC, Z-ER-AMC, Abz-GIRRAK(Dnp)-OH, Abz-GnVRAK(Dnp)-OH (“n” represents norleucine), and Abz-GIVRAK(Dnp)-NH_2_ were custom synthesized by GenScript (Piscataway, NJ). Z-RR-AMC was purchased from Bachem (#4004789) (Torrance, CA), and Abz-GIVRAK(Dnp)-OH was purchased from Bachem (#4049308) (Torrance, CA). Z-FR-AMC was purchased from Anaspec (#AS-24096) (Fremont, CA). Abz-GIERAK(Dnp)-OH was custom synthesized by the Wolan Lab at TSRI (La Jolla, CA). Assays were conducted at room temperature in triplicate, and relative fluorescence readings (RFUs) (excitation 320 nm, emission 400 nm for internally quenched substrates and excitation 360 nm, emission 460 nm for peptide-AMC substrates) were recorded over a period of 30 min by a Biotek HTX microplate plate reader. Enzyme velocity (RFU/sec) was calculated using the highest slope recorded for 10 consecutive fluorescent readings within the initial 30 min, and the mean and standard deviation (SD) were determined from the three technical replicates. RFU/s were converted to specific activity pmol/min/μg using AMC standards. For peptide-AMC substrates, specific activity was defined as AMC pmol/min/μg enzyme. To convert RFU/s to pmol/min for the internally quenched substrates Abz-GIVRAK(Dnp)-OH, Abz-GnVRAK(Dnp)-OH, Abz-GIERAK(Dnp)-OH, Abz-GIRRAK(Dnp)-OH, and Abz-GIVRAK(Dnp)-NH_2_, these substrates of 40–0.02 μM were serially diluted and incubated with excess cathepsin B (160 ng/μL) to generate a standard curve using the total fluorescence values detected at each concentration.

The kinetic parameters of *k*_cat_ and *K*_m_ for Abz-GIVRAK(Dnp)-OH and Abz-GIVRAK(Dnp)-NH_2_ substrates were determined at pH 4.6, pH 5.5, and pH 7.2, using substrate concentrations of 80–0.9 μM with 0.04 ng/μL cathepsin B for Abz-GIVRAK(Dnp)-OH and 0.40 ng/μL of cathepsin B for Abz-GIVRAK(Dnp)-NH_2_. RFU values were converted to s^–1^ using standard curves obtained from full digestion of Abz-GIVRAK(Dnp)-OH and Abz-GIVRAK(Dnp)-NH_2_ by using excess cathepsin B (160 ng/μL). The *k*_cat_ and *K*_m_ values were obtained from curve fitting the converted RFU data to the equation *v*_0_ = *V*_max_[*S*]/(*K*_m_ + [*S*]) where v_0_ is the activity at its corresponding substrate concentration [*S*] and *V*_max_ is the maximum enzyme velocity at saturated [*S*] concentration. *V*_max_ = *k*_cat_[*E*]_T_, where [*E*]_T_ is the total cathepsin B concentration. *K*_m_ is the *x*-axis value (substrate concentration) where *y* = *V*_max_/2. SD values for *k*_cat_ and *K*_m_ were determined from curving fitting the *v*_0_ and [*S*] data from three technical replicates. All data were plotted, calculated, and analyzed using GraphPad Prism9 software.

### Protease Cleavage Profiling by MSP-MS

MSP-MS was performed for cathepsin B, as well as cathepsin K, at pH 4.6 and pH 7.2. In a total volume of 22 μL, cathepsin B (0.1 ng/μL) or cathepsin K (0.07 ng/μL) were incubated with a mixture of 228 14-mer peptides (0.5 μM for each peptide) in assay buffer composed of 50 mM citrate phosphate at pH 4.6 or pH 7.2, 1 mM EDTA, 100 mM NaCl, and 4 mM DTT for 15 and 60 min at 25 °C. 10 μL was removed at 15 and 60 min and was combined with 60 μL of 8 M urea. A control assay used cathepsin B and cathepsin K in each assay buffer mixed with 8 M urea for 60 min at 25 °C for inactivation, prior to addition of the peptide library. Assays were conducted in quadruplicate technical replicates. Samples were acidified by addition of 40 μL of 2% TFA, enriched, and desalted using custom-made C18 spin tips, evaporated to dryness in a vacuum centrifuge, and placed at −70 °C. Samples were resuspended in 40 μL of 0.1% TFA (solvent A), and 4 μL was used for LC–MS/MS using the method described previously.^18^

MS/MS data analysis was performed using PEAKS (v 8.5) software (Bioinformatics Solutions Inc.). MS2 data were searched with the 228-member 14-mer library sequence as the database, and a decoy search was conducted with peptide amino acid sequences in reverse order. A precursor tolerance of 20 ppm and 0.01 Da for MS2 fragments was defined. No protease digestion was specified. Data were filtered to 1% peptide and protein level false discovery rates with the target-decoy strategy. Peptides were quantified with label-free quantification and data normalized by TIC. Outliers from replicates were removed by Dixon’s Q testing. Missing and zero values were imputed with random normally distributed numbers in the range of the average of smallest 5% of the data ± SD. The control 0 min values in MSP-MS obtained for cathepsin B at pH 4.6 and pH 7.2 and cathepsin K at pH 4.6 were also analyzed by PEAKS (for *n* = 12). ANOVA testing was performed for peptide data of control 0, 15, and 60 min incubation conditions; those with *p* < 0.05 were considered for further analysis. Criteria for identification of cleaved peptide products included those with intensity scores of 8-fold or more above the quenched inactive cathepsins, evaluated by log_2_(active/inactivated enzyme) ratios for each peptide product with *p* < 0.05 by the two-tailed homoscedastic *t*-test.

Evaluation of the frequencies of the P4 to P4′ amino acids adjacent to the cleavage sites was conducted using the iceLogo software 1.3.8 where the “experimental data set” consisted of the detected cleavage sites and the “reference data set” consisted of all 2965 possible cleavages within the 228 MSP-MS library. Analyses involved *z*-scores calculated by the equation (*X* – μ)/σ, where *X* is the frequency of the amino acid in the “experimental data set”, μ is the frequency of a particular amino acid at a specific position in the “reference data set”, and σ is the SD. *z*-scores were utilized to generate iceLogo illustrations of the relative frequencies of amino acid residues at each of the P4 to P4′ positions of the cleaved peptides where heights of the single letter amino acids represent “percent difference”, defined as the difference in frequency for an amino acid appearing in the “experimental data set” relative to the “reference data set”. Ranked preferred amino acids are shown above the midline, and unpreferred amino acid differences are represented below the midline using *p* < 0.30 cutoff criteria in the iceLogo software.

### Cathepsin B Cleavage of DPCP Peptide Substrates Determined by Mass Spectrometry

Cathepsin B (0.148 ng/μL) was incubated in a total volume of 30 μL with 40 μM substrate (Abz-GIVRAK(Dnp)-OH, Abz-GIERAK(Dnp)-OH, and Abz-GIRRAK(Dnp)-OH, Abz-GnVRAK(Dnp)-OH or Abz-GIVRAK(Dnp)-NH_2_) in assay buffer consisting of 40 mM citrate phosphate at pH 4.6 or pH 7.2, 1 mM EDTA, 100 mM NaCl, and 5 mM DTT for 15 and 240 min at 25 °C. After incubation at the indicated time, 10 μL aliquots were removed and combined with 60 μL of 8 M urea. A control inactive cathepsin B assay consisted of cathepsin B in assay buffer combined with 8 M urea for 60 min at 25 °C, prior to addition of substrate. After incubation, collected samples were acidified by addition of 40 μL of 2% TFA, desalted using C18 spin tips, evaporated to dryness in a vacuum centrifuge, and stored at −70 °C. Samples were resuspended in 400 μL of 0.1% TFA (solvent A) and 1 μL was used for LC–MS/MS analysis. Determination of peptide products for cleavage site analysis was performed by searching MS1 data for predicted *m/z* values of peptide cleavage products (Table S1).

## Results and Discussion

### Results

#### pH Profile of Cathepsin B Activity with Abz-GIVRAK(Dnp)-OH DPCP and Z-RR-AMC Endopeptidase Substrates

**Figure 1:**
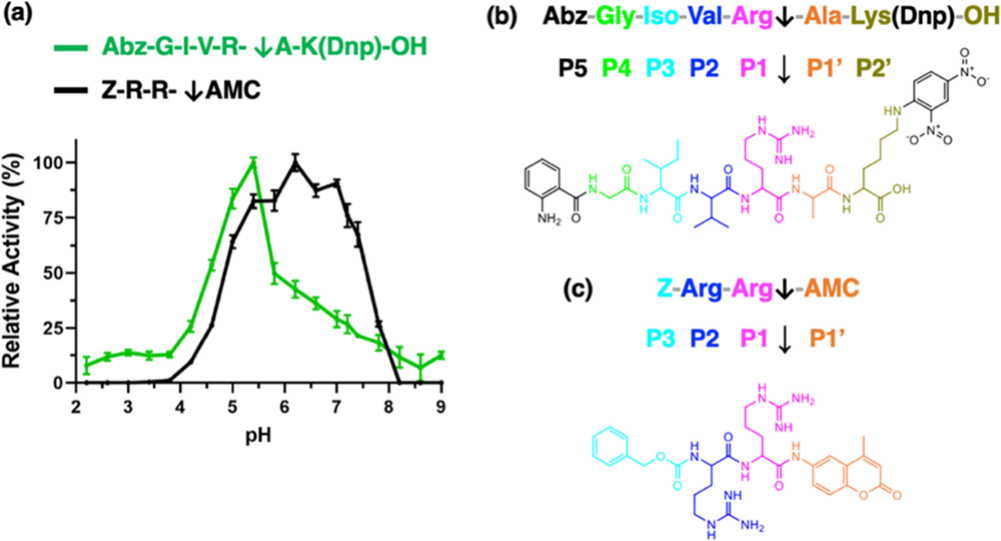
Cathepsin B activity monitored with Abz-GIVRAK(Dnp)-OH and Z-RR-AMC substrates under acidic to neutral pH conditions. (a) Acidic and neutral pH properties of cathepsin B monitored with Abz-GIVRAK(Dnp)-OH and Z-RR-AMC substrates. The pH-dependent cleavage activity of cathepsin B for the substrates Abz-GIVRAK(Dnp)-OH and Z-RR-AMC was assessed at pH 2.2–9.0, with substrate concentrations of 40 μM. Cathepsin B specific activity for each substrate has been normalized to its maximum activity detected across all pH conditions. Data are shown as the mean ± SD (standard deviation) of technical triplicates (*n* = 3) and this experiment was conducted three times. Cathepsin B activities at the pH values tested were significantly different from that of the enzyme’s optimum pH of 5.4 (*p* < 0.05, Student’s *t*-test). (b) Abz-GIVR↓AK(Dnp)-OH residues adjacent to the P1-P1′ cleavage site. The structure of the Abz-GIVR↓AK(Dnp)-OH substrate showing the amino acid residues at the P5 to P2′ positions of the P1↓P1′ cleavage site is illustrated. (c) Z-RR-↓AMC residues at the P1-P1′ cleavage site. The structure Z-RR-↓AMC illustrates the amino acid residues at the P3 to P1′ positions of the P1↓P1′ cleavage site.

Human cathepsin B activity is commonly measured with the Abz-GIVRAK(Dnp)-OH substrate for DPCP activity and the Z-RR-AMC substrate for endopeptidase activity. The pH profiles of these two substrates were directly compared, and data showed that cathepsin B cleaved Abz-GIVR↓AK(Dnp)-OH with highest efficiency at pH 5.4, with ≥50% of maximal activity observed at pH 4.6 to pH 5.8 (Figure 1a). We confirmed by nano-LC–MS/MS tandem mass spectrometry that cleavage of Abz-GIVR↓AK(Dnp)-OH occurred between R↓A to yield the product AK(Dnp)-OH (Figure S1). In contrast, cleavage of Z-RR-AMC was highest at pH 6.2 and displayed ≥50% of maximal activity at pH 5.0 to pH 7.4 (Figure 1a). Illustration of Abz-GIVR↓AK(Dnp)-OH cleavage occurring between R↓A indicates the P1↓P1′ cleavage site with adjacent residues which separate the fluorescent reporter group (Abz) and the quencher group (Dnp) (Figure 1b). Cleavage of Z-RR-AMC occurs between R-↓AMC with P1 as the Arg residue to release the free fluorescent AMC reporter (Figure 1c).

These pH profiles show that cathepsin B cleavage of the DPCP substrate Abz-GIVR↓AK(Dnp)-OH occurred preferentially in the acidic range of pH 4.6–5.8, whereas cleavage of Z-RR-AMC, an endopeptidase substrate, occurred in a more neutral pH range of pH 5.0–7.4. Cathepsin B cleaves numerous diverse substrates and, thus, it is not known whether other diverse peptide substrates show the same pH profiles as the Abz-GIVR↓AK(Dnp)-OH DPCP substrate and the Z-RR-AMC endopeptidase substrate. Therefore, the pH profiles of a diverse set of peptide substrates were assessed for DPCP and endopeptidase activities of cathepsin B under acidic and neutral pH conditions using the approach of MSP-MS.

#### Cleavage Profiling of Cathepsin B by MSP-MS Reveals That the Major DPCP and Minor Endopeptidase Activities Both Occur at Acidic and Neutral pH Conditions, with pH-dependent Cleavage Properties

MSP-MS protease cleavage profiling utilized a library of 228 14-mer peptides that can be cleaved by endopeptidases and exopeptidases. Each peptide has a free carboxyl terminus that can be cleaved by carboxypeptidases^42,47^ and a free amino terminus that is cleavable by aminopeptidases.^42,48,49^ Furthermore, the peptides can be cleaved by endopeptidases at sites that are distal from the N- and C-termini.^41,43^ Cathepsin B was incubated with the peptide library at pH 4.6 and pH 7.2, and cleavage products were identified and quantitated by LC–MS/MS tandem mass spectrometry. In parallel, the same peptide library was incubated with human cathepsin K at pH 4.6 and pH 7.2; cathepsin K was chosen for this comparative study because it is (1) a homologous cysteine cathepsin protease of cathepsin B and (2) active across a broad pH range and (3) has been validated as a strict endopeptidase.^45^

**Figure 2:**
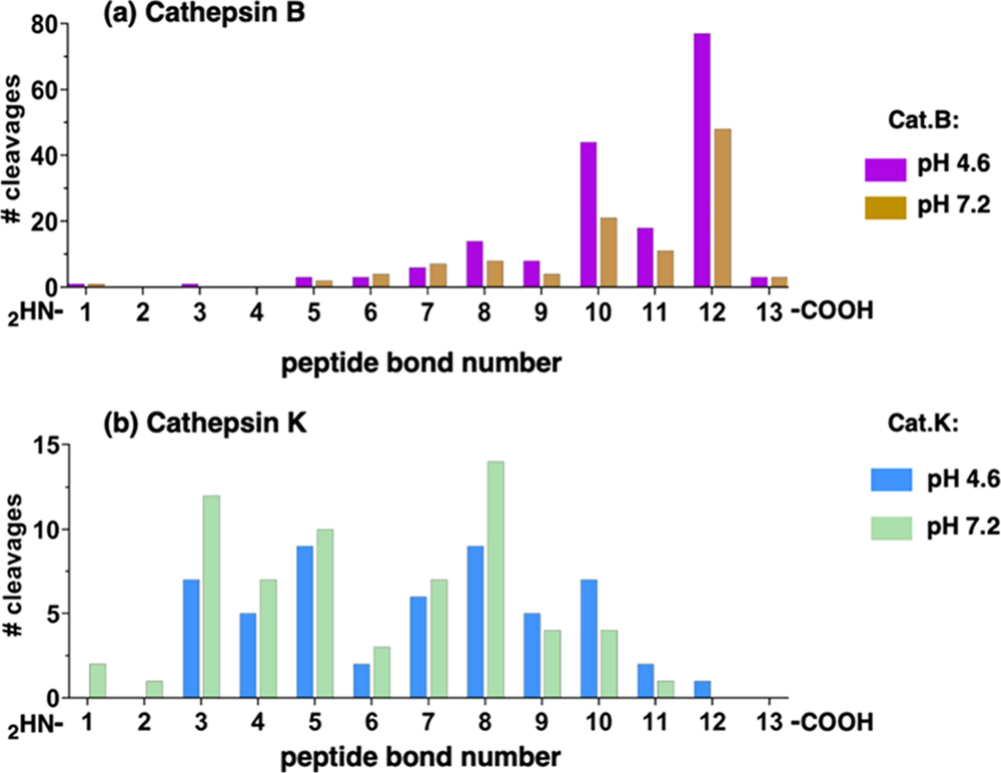
Cleavage profiling of cathepsin B DPCP and endopeptidase activities by MSP-MS conducted at acidic pH 4.6 and neutral pH 7.2. (a) Cathepsin B MSP-MS under acidic and neutral pH conditions demonstrates major DPCP activity. Cathepsin B was subjected to MSP-MS analysis of exopeptidase and endopeptidase cleavage sites at pH 4.6 and pH 7.2 (using four technical replicates). The number of cleavages at peptide bonds #1–13 of the diverse library of 228 peptides after incubation for 15 min was determined as described in the methods. At pH 4.6, 179 cleavages were assessed in the plotted graph for the number of cleavages at each peptide bond location. At pH 7.2, 109 cleavages of the peptide library are graphed similarly. This MSP-MS experiment was conducted two times. (b) Cathepsin K MSP-MS under acidic and neutral pH conditions demonstrates major endopeptidase activity. Cathepsin K was subjected to MSP-MS analysis at pH 4.6 and pH 7.2. The number of cleavages at each of the peptide bonds #1–13 of the peptide library generated after 15 min incubation was determined as described in the methods using four technical replicates.

In the pH 4.6 MSP-MS assay, cathepsin B cleaved the peptide library at 179 sites with the majority occurring at peptide bond #12 (Figure 2a). At pH 7.2, cathepsin B (at the same concentration as pH 4.6) cleaved at 109 sites with the majority also occurring at peptide bond #12 (Figure 2a). Cleavage at peptide bond #12 releases dipeptides from the C-terminus and thereby validates the strong DPCP activity of this enzyme. Cleavage at other sites is due to either sequential release of a dipeptide (e.g., positions #10, #8 and #6) or endopeptidase activity (e.g., position #5, #7, #9 and #11). To contrast these properties of cathepsin B with the cleavage site profile of an endopeptidase, we show that cathepsin K displays solely endopeptidase activity by cleavage at positions 3–10 (Figure 2b). Significantly, MSP-MS analysis of cathepsin B showed that major DPCP and minor endopeptidase cleavages occur under both acidic pH 4.6 and neutral pH 7.2 conditions of the 14-mer peptide substrates. The MSP-MS assay advantageously distinguishes between endopeptidase and DPCP activities (Figure 2).

**Figure 3:**
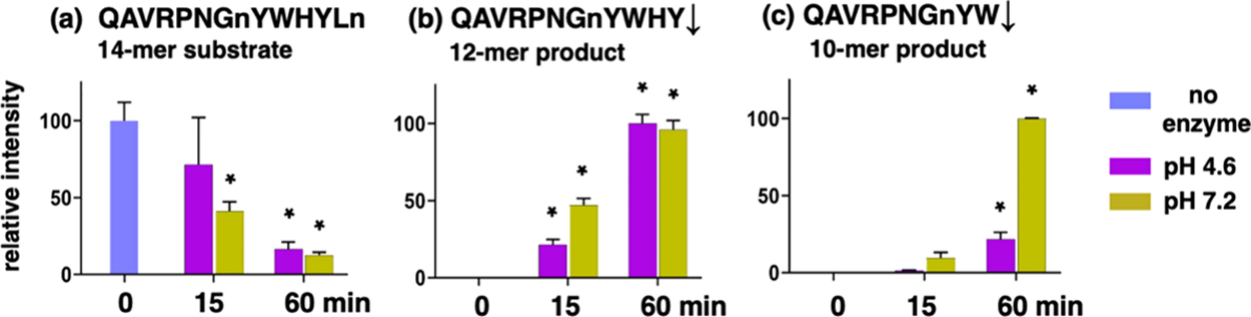
Sequential DPCP activity demonstrated by the time-dependent conversion of a 14-mer peptide substrate to 12-mer and 10-mer peptide products. The 14-mer substrate QVRPNGnYWYLn (panel a), 12-mer product QAVRPNGnYWHY (panel b), and the 10-mer product QAVRPNGnYW (panel c) were quantitated at time points of 15- and 60-min incubation times (using four technical replicates). This experiment was conducted two times. Norleucine is represented by “n”. Data are shown as the mean ± SEM (standard error of the mean) of four technical replicates (*n* = 4). Values at 15 min or 60 min that are significantly different from the “0” time at each pH are indicated by asterisks (*) (*p* < 0.05 by Student’s *t*-test).

Cathepsin B DPCP cleavages at peptide bonds #12 and #10 may occur sequentially, as shown, for example, by conversion of the QAVRPNGnYWHYLn 14-mer peptide substrate to its 12-mer QAVRPNGnYWHY and 10-mer QAVRPNGnYW products in a time-dependent manner (Figure 3). Sequential DPCP cleavages of the peptide library substrates at peptide bond #12 followed by sequential DPCP cleavages would be indicated by products with cleavages occurring at peptide bonds #10 and #8 (Figure 2a).

#### MSP-MS Analysis of Cleavage Site Preferences by Cathepsin B Leads to Design of pH-Dependent Substrates for DPCP Activity

**Figure 4:**
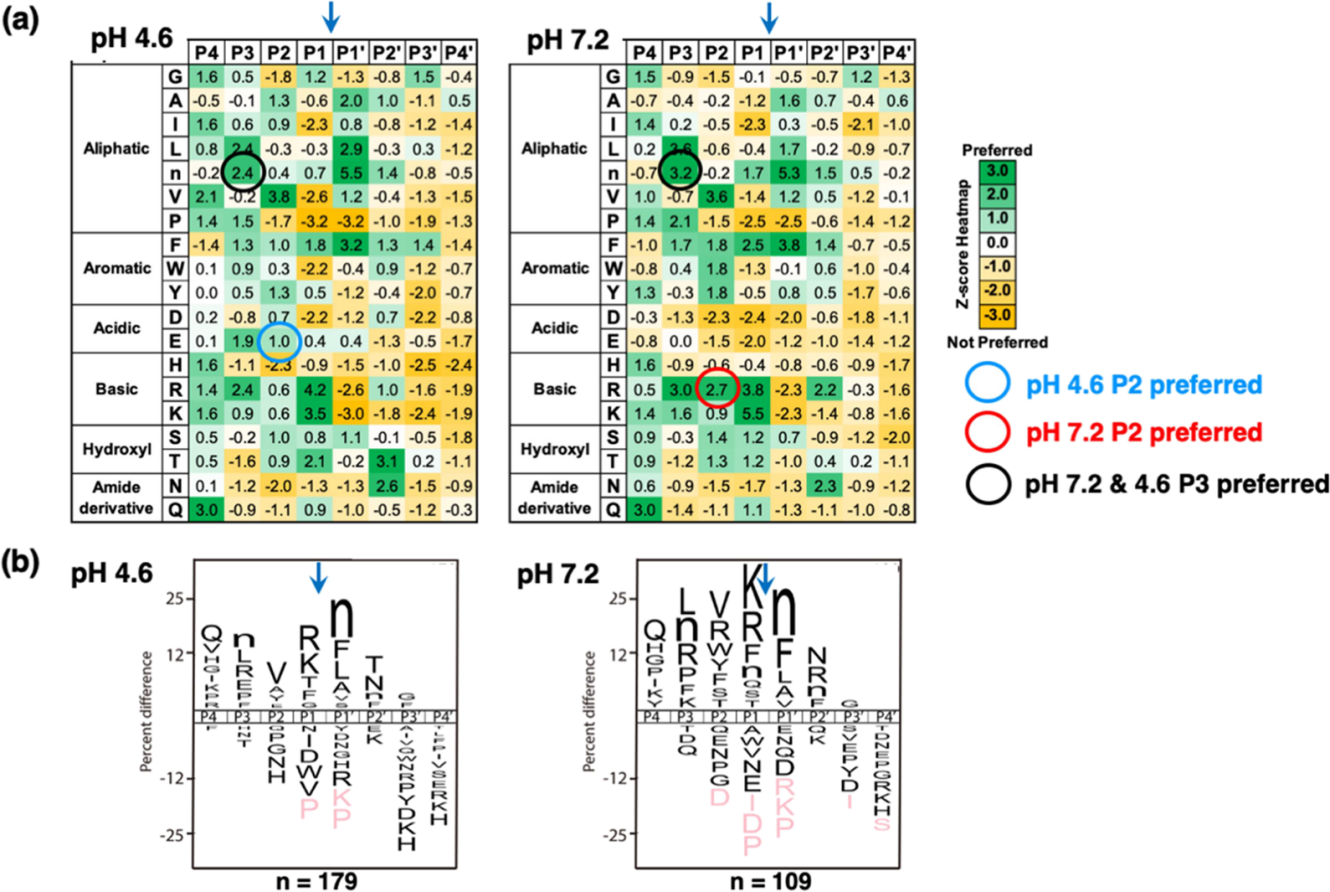
Preferred residues of cathepsin B peptide cleavages at pH 4.6 and pH 7.2 assessed by MSP-MS data. (a) Heatmap of *z*-scores for preferred amino acids adjacent to the cleavage sites at pH 4.6 and pH 7.2. Preferred amino acid residues at P4 to P4′ positions of the cleavage sites are shown for pH 4.6 and pH 7.2 as *z*-score analysis of MSP-MS cleavage profiling data as described in the methods. The experiment was conducted with four technical replicates, and the experiment was repeated two times. The heatmap of *z*-scores shows norleucine as “n.” (b) IceLogo illustration of the primary residues adjacent to cleavage sites. MSP-MS data were analyzed by iceLogo (based on *z*-scores) to show the relative frequency of residues at the P4 to P4′ positions of peptide cleavage sites. Relative to the cleavage site, amino acids shown above the mid-line are preferred amino acids by cathepsin B and those below the mid-line are amino acids not preferred by cathepsin B, based on comparison of experimental cleavage data and all possible products within the MSP-MS peptide library with *p* < 0.3 as described in the methods.

MPS-MS data characterized the preferred amino acid residues at the P4 to P4′ positions adjacent to the P1↓P1′ cleavage sites at acidic pH 4.6 compared to neutral pH 7.2. *z*-scores provided quantitative comparison of preferred and nonpreferred residues at the P4 to P4′ positions illustrated by heatmaps (Figure 4a). The heatmaps were used to assess differences in preferred residues under the two pH conditions for the goal of designing pH-selective substrates for DPCP activity of cathepsin B. More specifically, we focused on pH differences at the P2 position because several preferred residues at this position differed at pH 4.6 compared to pH 7.2 (Figure 4a).

At pH 4.6, Glu at the P2 position was preferred with a *z*-score of 1.0, but Glu was not preferred at pH 7.2 shown by the *z*-score of −1.5 (Figure 4a). These *z*-scores had a difference of 2.5 which was the largest among preferred residues at the P2 position for pH 4.6. These data predicted that incorporation of Glu into the substrate Abz-GIER↓AK(Dnp)-OH may generate an acid pH-selective substrate of DPCP activity.

At pH 7.2, Arg at the P2 position was preferred with a *z*-score of 2.7, but at pH 4.6, Arg at P2 had a smaller *z*-score of 0.6 (Figure 4a). The difference in the *z*-score of 2.1 for Arg at pH 7.2 and pH 4.6 suggested that Arg at P2 may provide a neutral pH-selective substrate. This finding suggested that inclusion of Arg in the substrate Abz-GIRR↓AK(*Dnp*)OH may provide a neutral pH-selective substrate.

The standard DPCP substrate of Abz-GIVR↓AK(*Dnp*)OH contains isoleucine at P3, valine at P2, and arginine at the P1 position. With respect to the P2 and P1 residues, there are strong preferences by cathepsin B for Val at the P2 residue at both pH 4.6 (*z*-score of 3.8) and pH 7.2 (*z*-score of 3.6) and stronger preference for Arg by cathepsin B at the P1 residue at pH 4.6 (*z*-score of 4.2) compared to pH 7.2 (*z*-score of 3.8). However, for the P3 residue, there is a weak preference for isoleucine at pH 7.2 (*z*-score of 0.2). However, norleucine at P3 is highly preferred at pH 4.6 (*z*-score of 2.4) and pH 7.2 (*z*-score of 3.2) (Figure 4a), which is also depicted by iceLogo (Figure 4b). It was, therefore, predicted that Abz-GnVR↓AK(*Dnp*)OH would display DPCP activity of cathepsin B under neutral pH as well as under acidic pH conditions, as shown by data described in the next section.

#### pH-Dependent Substrates for DPCP Activity of Cathepsin B

**Figure 5:**
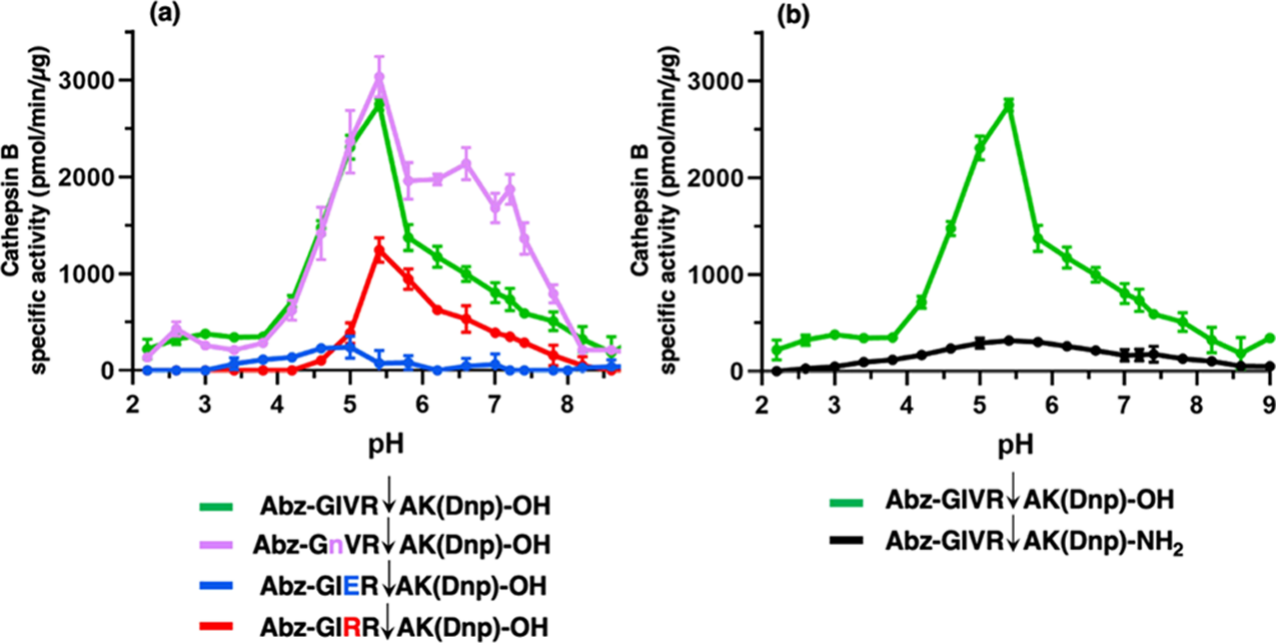
Cathepsin B displays DPCP activity under acidic to neutral pH conditions, illustrated by pH-dependent peptide substrates. (a) Analysis of the pH-dependence of cathepsin B with DPCP substrates containing variant P2 residues, and pH-independence of a substrate with a variant P3 residue. The Abz-GIVR↓AK(Dnp)-OH typically used in the field (24) to monitor cathepsin B DPCP activity was modified at the P2 position to generate Abz-GIRR↓AK(Dnp)-OH and Abz-GIER↓AK(Dnp)-OH substrates and then was assessed at pH 2–9 for DPCP activity. The substrate Abz-GnVRAK(Dnp)-OH with variant residue at the P3 position was also assessed at pH 2–9 for DPCP activity. Data are shown as the mean ± SD (standard deviation) of three technical replicates (*n* = 3), and this experiment was repeated three times. Cleavage of these substrates between R↓A occurred to generate AK(Dnp)-OH was confirmed by mass spectrometry (Figures S1–S4). (b) Comparison of cathepsin B activity with the DPCP substrate Abz-GIVRAK(Dnp)-OH and the endopeptidase substrate Abz-GIVRAK(Dnp)-NH_2_ at pH 2–9. Cathepsin B activity was monitored with the Abz-GIVRAK(Dnp)-OH and Abz-GIVRAK(Dnp)-NH_2_ substrates for DPCP and endopeptidase activity was assessed at pH 2–9. Data points are the averages from 3 replicate assays. Data are shown as the mean ± SD (standard deviation) of three technical replicates (*n* = 3), and this experiment was repeated three times. Cleavage at R↓A to generate AK(Dnp)-NH_2_ was confirmed by mass spectrometry (Figure S5). For Abz-GIVRAK(Dnp)-OH (panel a and b), Abz-GnVRAK(Dnp)-OH (panel a), and Abz-GIRRAK(Dnp)-OH (panel a) substrates, cathepsin B activity with these substrates was optimal at pH 5.4 and the other pH values were significantly different from that of pH 5.4 (*p* < 0.05, Student’s *t*-test). For the Abz-GIERAK(Dnp)-OH substrate (panel a), optimal activity was observed at pH 5.0 with >50% reduction at pH 3.8 and pH 5.4. For Abz-GIVRAK(Dnp)-NH_2_ substrate (panel b), optimal activity was observed at pH 5.4 with a plateau of similar activity at pH 4.6–6.2, and significantly lower activity at pH 4.2 or below and at pH 6.6 or above (with the exception of pH 7.4) (*p* < 0.05, Student’s *t*-test).

Novel substrates for DPCP activity of cathepsin B predicted to be selective for acidic pH or neutral pH conditions, based on MSP-MS *z*-score data, were demonstrated (Figure 5a). Cleavage of the substrate Abz-GIER↓AK(Dnp)-OH occurred at acidic pH 4.0–5.0 and demonstrated no detectable DPCP activity above pH 6.2. In contrast, cleavage of the substrate Abz-GIRR↓AK(Dnp)-OH occurred in a higher pH range of pH 5–7 and demonstrated no detectable DPCP activity by cathepsin B under acidic pH conditions below pH 4.6. Furthermore, the substrate Abz-GnVR↓AK(Dnp)-OH detected enhanced DPCP activity of cathepsin B under both acidic and neutral pH conditions compared to the original substrate Abz-GIVR↓AK(Dnp)-OH (Figure 5a), especially toward neutral pH. Thus, the cleavage profiling data obtained by MSP-MS (Figure 4) provided design and demonstration of pH-dependent DPCP substrates as well as Abz-GnVR↓AK(Dnp)-OH displaying a broad pH range for detecting DPCP activity of cathepsin B (Figure 5a). Cleavage of all these DPCP substrates was verified to occur between R↓A to generate AK(Dnp)-OH demonstrated by mass spectrometry (Figures S2–S4).

#### Comparison of Cathepsin B DPCP and Endopeptidase Activities Monitored with Abz-GIVRAK(Dnp)-OH and Abz-GIVRAK(Dnp)-NH_2_ Substrates, Respectively

To evaluate the effect of the substrate C-terminal carboxylate on cathepsin B’s pH-dependent DPCP activity, the Abz-GIVR↓AK(Dnp)-OH substrate for DPCP activity was modified with a C-terminal amide to generate the endopeptidase substrate Abz-GIVR↓AK(Dnp)-NH_2_. Assessment of cathepsin B activity with these two substrates at pH 2–9 showed that the DPCP activity monitored by Abz-GIVR↓AK(Dnp)-OH was greater than the endopeptidase activity monitored by Abz-GIVR↓AK(Dnp)-NH_2_, with a 12-fold difference at the optimal pH of 5.4 (Figure 5b). The endopeptidase activity was lower than the DPCP activity across the entire pH range of 2–9. Cathepsin B cleavages of Abz-GIVRAK(Dnp)-OH and Abz-GIVRAK(Dnp)-NH_2_ substrates between R↓A was confirmed by mass spectrometry of peptide products (Figures S1 and S5).

Kinetic constants of the DPCP activity monitored with the Abz-GIVRAK(Dnp)-OH substrate and the endopeptidase activity monitored with the Abz-GIVRAK(Dnp)-NH_2_ substrate were compared under the pH conditions of pH 4.6 representing lysosomes,^22,23^ pH 5.5 representing secretory vesicles,^42^ and pH 7.2 representing cytosol, nuclei, and extracellular locations of cathepsin B^20,21^ (Table 1). The DPCP specific activity was 7 times greater than the endopeptidase activity at pH 5.5. DPCP activity was also substantially greater than the endopeptidase activity at each pH condition.

**Table 1:** Kinetic Evaluation of Cathepsin B Activity with Abz-GIVRAK(Dnp)-OH and Abz-GIVRAK(Dnp)-NH_2_ Substrates for DPCP and Endopeptidase Activities, Respectively^a^

substrate	pH	*K*_m_ (μM)	*k*_cat_ (s^–1^)	*k*_cat_/*K*_m_ (s^–1^ mM^–1^)	specific activity (pmol/min/μg)
Abz-GIVRAK(Dnp)-OH (DPCP)	4.6	15 ± 2.3	4.2 ± 0.2	280	1500
	5.5	51 ± 8.6	7.4 ± 0.7	150	2800
	7.2	156 ± 64	2.3 ± 0.7	15	730
Abz-GIVRAK(Dnp)-NH_2_ (endopeptidase)	4.6	25 ± 3.7	0.4 ± 0.02	16	290
	5.5	40 ± 5.6	0.4 ± 0.03	10	390
	7.2	53 ± 14	0.2 ± 0.04	3.8	200

a Abz-GIVRAK(Dnp)-OH and Abz-GIVRAK(Dnp)-NH_2_ substrates for cathepsin B DPCP activity were characterized for kinetic values at pH 4.6, 5.5, and 7.2 as described in the methods.

The kinetic constants *k*_cat_/*K*_M_ and *k*_cat_ were higher for DPCP compared to endopeptidase activity at pH 4.6 and pH 5.5, but at pH 7.2. With respect to *K*_M_ values, the DPCP activity showed values of 15 and 51 μM at pH 4.6 and pH 5.5, respectively, but DPCP activity showed a higher *K*_M_ value of 156 μM at pH 7.2. The endopeptidase activity at pH 4.6, pH 5.5, and pH 7.2 displayed *K*_M_ values of 25, 40, and 53 μM, respectively.

These data show the importance of the C-terminal carboxylate of Abz-GIVRAK(Dnp)-OH for DPCP activity of cathepsin B because replacement with the amide group of Abz-GIVRAK(Dnp)-NH_2_ resulted in endopeptidase activity that was substantially lower than the DPCP activity.

#### pH Profiles of Endopeptidase Activities of Cathepsin B Monitored with Peptide-AMC Substrates

**Figure 6:**
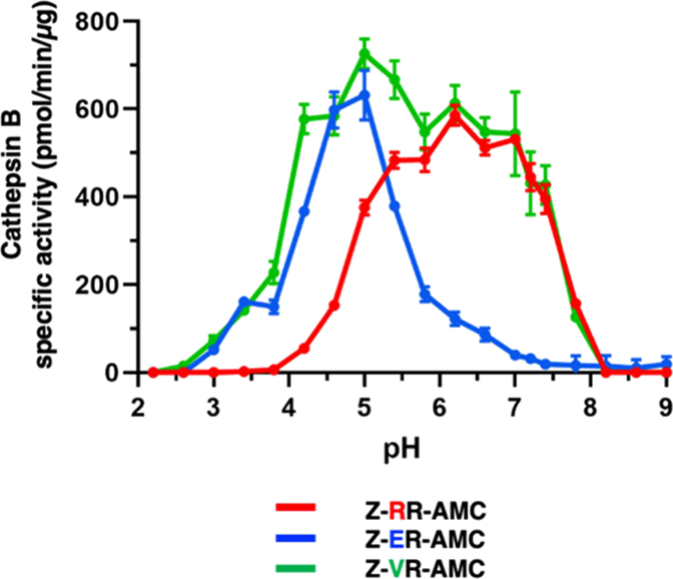
pH-dependent cathepsin B endopeptidase substrates illustrated by Z-RR-AMC, Z-ER-AMC, and Z-VR-AMC at pH 2–9. Cathepsin B activity was evaluated at pH 2–9 with peptide-AMC substrates having variant P2 residues consisting of Z-RR-AMC, Z-ER-AMC, and Z-VR-AMC. Data are shown as the mean ± SD (standard deviation) of triplicates (*n* = 3). For Z-RR-AMC, cathepsin B activity was optimal at pH 6.2 and activity at other pHs were significantly lower (*p* < 0.05, student’s *t*-test). For Z-ER-AMC, cathepsin B activity was optimal at pH 6.2 and activity at other pHs were significantly lower (*p* < 0.05, student’s *t*-test). For Z-VR-AMC, cathepsin B activity was optimal at pH 5.0 and activity was significantly lower at pH 4.6 and below, at pH 5.8 to pH 6.6, and at pH 7.2 and above (*p* < 0.05, student’s *t*-test).

Endopeptidase activity was evaluated with the peptide-AMC substrates Z-RR-AMC, Z-ER-AMC, and Z-VR-AMC with alterations of the P2 residues at acid to neutral pH conditions (Figure 6). Also, these peptide-AMC substrates were compared to the parallel Abz-GIRRAK(Dnp)-OH, Abz-GIERAK(Dnp)-OH, and Abz-GIVRAK(Dnp)-OH substrates (Figure 5a). Z-RR-AMC with Arg as the P2 residue displayed cathepsin B activity over the range of pH 5.0 to neutral pH 7.5, similar to that of Abz-GIRRAK(Dnp)-OH. Z-ER-AMC with Glu as the P2 residue displayed acidic pH cathepsin B activity at pH 4.5–5.5, similar to Abz-GIERAK(Dnp)-OH. Z-VR-AMC with Val as the P2 residue displayed optimal activity at pH 5.0, with activity occurring at pH 4–7, which had a broad pH profile more similar to Abz-GIVRAK(Dnp)-NH_2_ than Abz-GIVRAK(Dnp)-OH (Figure S6).

Despite similar pH profiles between the parallel DPCP and endopeptidase substrates, all the endopeptidase substrates had lower specific activity than each of their DPCP substrate counterparts. These differences may be explained by the lack of a C-terminal carboxylate group on these Z-peptide-AMC substrates that would otherwise promote favorable interactions with cathepsin B (Figures 5b and S6). These data support the MSP-MS findings that cathepsin B displays prominent DPCP activity over endopeptidase activity under both acidic and neutral pH conditions. However, endopeptidase activity can still be demonstrated across all pH conditions with activity being defined by amino acids in the P2 position. Taken together, these studies provide insight for Z-RR-AMC with Arg as the P2 residue as being poorly cleaved by cathepsin B at acidic pH, but activity is greatly improved by substituting either Glu or Val for Arg at P2.

**Figure 7:**
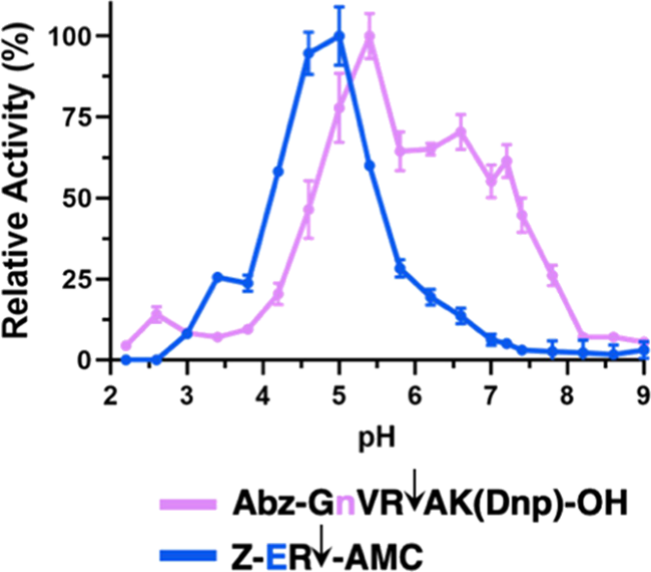
Cathepsin B DPCP activity at neutral pH monitored with the Abz-GnVRAK(Dnp)-OH substrate possessing a broad pH range for activity, and endopeptidase activity at acidic pH monitored with the pH-dependent substrate Z-ER-AMC. We developed novel substrates illustrating the concept that the Abz-GnVRAK(Dnp)-OH substrate monitors cathepsin DPCP activity at neutral pH with a broad pH profile for activity, and the acid pH-dependent substrate Z-ER-AMC monitors cathepsin B at acidic pH. Assays were conducted in triplicate; the mean ± SD are shown. These data are shown from Figures 5 and 6 in a normalized manner by maximum activity observed with each substrate.

Cathepsin B’s DPCP activity at neutral pH can be readily detected with the novel Abz-GnVRAK(Dnp)-OH substrate, and cathepsin B’s endopeptidase activity at acidic pH can be readily detected with the Z-ER-AMC substrate. New data from this study support adjustment of the prior view in the field that cathepsin B’s DPCP activity is higher at acidic pH and decreases at neutral pH (Figure 1),^26^ because here we showed that the DPCP activity monitored with Abz-GnVR↓AK(*Dnp*)OH displayed activity across a broad pH range (Figure 7). These data illustrate the new concept that DPCP activity of cathepsin B occurs under acidic and neutral pH conditions.

While Z-RR-AMC cleaving activity of cathepsin B has been reported to occur optimally at neutral pH and poorly at acidic pH (Figure 1),^28,30−32^ this study shows that Z-ER-AMC has the converse properties to monitor high endopeptidase activity of cathepsin B at acidic pH (Figure 7). These findings illustrate the new concept that the endopeptidase activity of cathepsin B occurs under acidic to neutral pH conditions.

Significantly, these data demonstrate that the pH-dependent cleavage of peptide substrates by cathepsin B under acidic to neutral pH conditions is based on the sequence of the substrate for both DPCP and endopeptidase activities of the enzyme.

## Discussion

Cathepsin B is a unique protease because it possesses dual DPCP and endopeptidase activities. While prior studies showed that this enzyme displays DPCP activity at acidic pH using the Abz-GIVRAK(Dnp)-OH substrate and endopeptidase activity a neutral pH using the Z-RR-AMC substrate,^31,35−39^ our recent finding that cathepsin B cleaves peptide substrates in a pH-dependent manner^18^ raises the question of whether the DPCP and endopeptidase activities may occur over a broad pH range represented by pH-dependent substrates rather than under restricted pH conditions. The answer was achieved in this study by global MSP-MS cleavage profiling of cathepsin B using a diverse library of 228 14-mer peptides. Importantly, MSP-MS cleavage results showed that the major DPCP and minor endopeptidase cleavages both occur under acidic and neutral pH conditions for cathepsin B. The proteolytic activities occurred from acidic to neutral pH conditions with pH-dependent substrates as well as substrates with broad pH activity profiles. These findings modify the prior view in the field of DPCP activity at acidic pH and endopeptidase activity at neutral pH^31,35−39^ to the new concept that the dual DPCP exopeptidase and endopeptidase activities of cathepsin B both occur under acidic to neutral pH conditions using pH-dependent peptide substrates.

The MSP-MS cleavage profiling data advantageously monitor DPCP and endopeptidase cleavages. DPCP cleavages remove C-terminal dipeptides from the 14-mer peptide substrates. These data demonstrated DPCP as the major type of proteolytic activity of cathepsin B, whereas the endoproteolytic cleavages occurred less frequently. These DPCP and endopeptidase cleavage properties of cathepsin B contrast with cathepsin K as a protease with only endopeptidase activity as shown by the MSP-MS assays. These MSP-MS data demonstrate the prominent DPCP and minor endopeptidase cleavages by cathepsin B.

Cleavage profiling data by MSP-MS provided design and demonstration of substrates with different pH profiles for the DPCP activity of cathepsin B. The novel DPCP substrate Abz-GnVR↓AK(Dnp)-OH utilized the preferred norleucine (n) residue at the P3 position and was found to effectively monitor DPCP activity of cathepsin B over a broad pH range, from acidic to neutral pH values, with high specific activity. The broad pH profile of the Abz-GnVRAK(Dnp)-OH substrate contrasts with the restricted acidic pH profile of the original Abz-GIVR↓AK(Dnp)-OH substrate. These findings demonstrate Abz-GnVR↓AK(Dnp)-OH as a useful substrate to monitor DPCP of cathepsin B over a broad pH range. Preferences for different P2 residues under acidic and neutral pH conditions led to development of Abz-GIRR↓AK(Dnp)-OH as a substrate that was cleaved by cathepsin B at pH 5–7. Abz-GIER↓AK(Dnp)-OH was designed and displayed activity at pH 4–5 representing acidic conditions. Thus, pH-dependent substrates for DPCP activity exist as illustrated by Abz-GIRR↓AK(Dnp)-OH and Abz-GIER↓AK(Dnp)-OH that display pH profiles in the neutral and acidic pH ranges, respectively. These findings demonstrate that modification of P3 and P2 residues of Abz-GIVR↓AK(Dnp)-OH leads to altered pH profiles of cathepsin B’s DPCP activity.

Cleavage profiling data facilitated the design and assessment of Z-peptide-AMC endopeptidase substrates with pH-selective preferences for P2 residues. The known Z-RR-AMC substrate, with Arg as the preferred P2 residue at neutral pH 7.2, is cleaved by cathepsin B activity in a more neutral pH range compared to acid pH conditions, consistent with MSP-MS data indicating Arg as a preferred P2 residue at neutral pH 7.2 compared to acidic pH 4.6. In contrast, the Z-ER-AMC substrate, with Glu as the preferred P2 residue at pH 4.6, preferentially monitored cathepsin B activity in the acid pH condition compared to neutral pH. Furthermore, Val as P2 residue is preferred at both acidic and neutral pH and, thus, the Z-VR-AMC substrate monitors cathepsin B activity over a broad pH range at acidic and neutral pH. These peptide-AMC substrates demonstrate pH-dependent as well as broad pH activity profiles for the endopeptidase activity of cathepsin B.

Comparison of the DPCP activity of cathepsin B monitored with the Abz-GIVRAK(Dnp)-OH substrate with C-terminal carboxylate compared to the endopeptidase substrate Abz-GIVRAK(Dnp)-NH_2_ with C-terminal amide further demonstrated the major DPCP activity with greater specific activity than the endopeptidase activity under acidic to neutral pH conditions. Like Abz-GIVRAK(Dnp)-NH_2_, the Z-VR-AMC endopeptidase substrate also displayed a broad pH profile for activity of lower specific activity than the DPCP substrate Abz-GIVRAK(Dnp)-OH. These data support the concept that the major DPCP activity and lesser endopeptidase activity of cathepsin B occur over a broad pH range.

Prior evaluation of the structural features of cathepsin B, conducted at pH 5.5, suggested that the unique occluding loop may participate in mediating the DPCP activity under acidic pH conditions, and alteration of the occluding loop at neutral pH may allow the active site to become accessible to endopeptidase substrates.^37,38^ The occluding loop corresponding to residues 102–128 contains two His residues (His110 and His111) that directly interact with the C-terminal carboxylic acid group.^50,51^ It has been viewed that the occluding loop accommodates DPCP substrates at acidic pH and may acquire a pose at neutral pH that allows accessibility to endopeptidase substrates. However, the ongoing view of the occluding loop should now be modified based on the findings that DPCP and endopeptidase cleavages of cathepsin B occur under both acidic and neutral pH conditions. The current data of this study suggest that the occluding loop allows both DPCP and endopeptidase substrates to bind cathepsin B at the active site for proteolysis. Changes in the configuration of the occluding loop may be predicted to participate in utilization of pH-dependent DPCP and endopeptidase substrates across acidic to neutral pH conditions. Several studies have shown that mutagenesis of the occluding loop domain alters the relative DPCP and endopeptidase activities of cathepsin B.^36,38^ It will be of interest in future studies to compare cathepsin B crystal structures under acidic and neutral pH conditions to gain understanding of the molecular features for substrate docking to achieve dual DPCP and endopeptidase activities of the enzyme.

## Conclusions

In summary, global cleavage profiling of cathepsin B with a diverse library of peptide substrates provided evidence that the primary DPCP and modest endopeptidase activities of cathepsin B both occur under acidic and neutral pH conditions. Significantly, MSP-MS data indicated norleucine (n) as a preferred residue at the P3 position at acidic and neutral pH conditions which led to design of the novel substrate Abz-GnVRAK(Dnp)-OH that efficiently monitors DPCP activity of cathepsin B over a broad pH range. The broad pH profile of Abz-GnVRAK(Dnp)-OH contrasts with the more restricted acidic pH profile of the original Abz-GIVRAK(Dnp)-OH substrate. Furthermore, modification of the P2 residue of the original Z-RR-AMC substrate demonstrated that the endopeptidase activity of cathepsin B can be readily detected at acidic pH with the Z-ER-AMC substrate, as well as with the Z-VR-AMC substrate that has a broader pH activity profile. These peptide-AMC substrates demonstrate that pH-dependent and broad pH activity profile substrates of cathepsin B exist for both its DPCP and endopeptidase activities. This study highlights the unique properties of cathepsin B having dual DPCP and endopeptidase activities covering biological pH conditions from lysosomal acidic pH to cytosolic and other neutral pH cellular conditions.

Cathepsin B cleavage of biological substrates in prior studies have illustrated the enzyme’s endopeptidase and exopeptidase activities under acidic to neutral pH conditions, consistent with findings of this study for the broad pH ranges of endopeptidase and DPCP cleavages by this protease. Cathepsin B endopeptidase activity cleaves biological substrates that include the propeptide region within pro-cathepsin for autoactivation to mature cathepsin B,^52^ thyroglobulin,^53,54^ MARCKS,^25^ invariant chain,^55^ secretory leucoprotease inhibitor,^56^ sphingosine kinase-1,^57^ collagen,^58^ amyloid precursor protein (APP) as a candidate β-secretase,^59,60^ and proneuropeptide substrates^42^ under pH conditions ranging from acidic lysosomal pH to mildly acidic secretory vesicles and neutral extracellular pH conditions. In addition, exopeptidase activity has also been detected using thyroglobulin and glucagon as substrates.^53,61^ Interestingly, different products were generated from collagen by cathepsin B cleavage that varied with pH conditions.^58^ We postulate that these observed differences in cleavage sites of biological substrates are due to pH-dependent amino acid preferences as demonstrated by the MSP-MS cleavage profiling data of this study. It will, therefore, be of interest in future studies to gain knowledge of *in vivo* substrates of cathepsin B at cellular locations of distinct pH conditions in health and disease.

## Supporting Information Available

DPCP peptide substrates and cleavage products generated by cathepsin B, mass spectrometry of DPCP substrate-derived cleavage products, and comparison of endopeptidase and DPCP substrates over a broad pH range (PDF)

## Notes

LC–MS/MS data files can be obtained through massive.ucsd.edu under the data set identifier numbers MSV000088932. Data analysis is provided in the methods and the Supporting Information.
