# Supplementary material for: Cathepsin B Dipeptidyl Carboxypeptidase and Endopeptidase Activities Demonstrated across a Broad pH Range
Source: Biochemistry. 2022 Aug 18;61(17):1904–14. doi: 10.1021/acs.biochem.2c00358 (PMC9454093; doi:10.1021/acs.biochem.2c00358)

Supporting Information

Cathepsin B dipeptidyl carboxypeptidase and endopeptidase activities demonstrated across a broad pH range

Michael C. Yoon[1, 2], *Vivian Hook[1-3], *Anthony J. O'Donoghue[1, 2]

[1]Skaggs School of Pharmacy and Pharmaceutical Sciences, University of California, San Diego, La Jolla, CA 92093; [2]Biomedical Sciences Graduate Program, University of California, San Diego, La Jolla, CA 92093; [3]Department of Neurosciences and Department of Pharmacology, School of Medicine, University of California, San Diego, La Jolla, CA 92093

List of Table and Figures:

Table S1. DPCP peptide substrates and cleavage products generated

Figure S1. Abz-GIVR↓AK(Dnp)-OH cleavage by cathepsin B generates AK(Dnp)-OH illustrated by mass spectrometry.

Figure S2. Abz-GIER↓AK(Dnp)-OH cleavage by cathepsin B generates AK(Dnp)-OH illustrated by mass spectrometry

Figure S3. Abz-GIRR↓AK(Dnp)-OH cleavage by cathepsin B generates AK(Dnp)-OH illustrated by mass spectrometry

Figure S4. Abz-GnVR↓AK(Dnp)-OH cleavage by cathepsin B generates AK(Dnp)-OH illustrated by mass spectrometry

Figure S5. Abz-GIVR↓AK(Dnp)-NH_2 cleavage by cathepsin B generates AK(Dnp)-NH_2 illustrated by mass spectrometry

Figure S6. Cathepsin B cleavage of the endopeptidase substrates Abz-GIVRAK(Dnp)-NH_2 and Z-VR-AMC compared to the DPCP substrate Abz-GIVRAK(Dnp)-OH.

Table S1. DPCP peptide substrates and cleavage products generated by cathepsin B.

peptide substrate	DPCP cleavage products	Exact mass	m/z (+1)
Abz-GIVR↓AK(Dnp)-OH	Abz-GIVR	562.32	563.327
	AK(Dnp)-OH	383.14	384.147
Abz-GnVR↓AK(Dnp)-OH	Abz-GnVR	562.32	563.327
	AK(Dnp)-OH	383.14	384.147
Abz-GIER↓AK(Dnp)-OH	Abz-GIER	592.30	593.307
	AK(Dnp)-OH	383.14	384.147
Abz-GIRR↓AK(Dnp)-OH	Abz-GIRR	619.36	620.367
	AK(Dnp)-OH	383.14	384.147
Abz-GIVR↓AK(Dnp)-NH_2	Abz-GIVR	562.32	563.327
	AK(Dnp)- NH_2	382.16	383.167

Masses of predicted DPCP cleavage products generated by cathepsin B from the indicated substrates are shown. Nano-LC-MS/MS tandem mass spectrometry identified the dipeptide product AK(Dnp)-OH and AK(Dnp)-NH_2. The Abz-tetrapeptide products were not well observed in the LC-MS/MS method. However, the dipeptide products demonstrate DPCP type of proteolytic activity of cathepsin B.

Figure S1. Abz-GIVR↓AK(Dnp)-OH cleavage by cathepsin B generates AK(Dnp)-OH illustrated by mass spectrometry. A time-course of cathepsin B incubation with Abz-GIVRAK(Dnp)-OH for 30 min and 240 min at pH 4.6 and pH 7.2 was analyzed by nano-LC-MS mass spectrometry. The TIC (total ion chromatogram) illustrates the retention time (RT) of the AK(Dnp)-OH cleavage product, panel a. Mass spectra for the DPCP cleavage product of AK(Dnp)-OH m/z (+1) of 384.15 is shown in panel b.

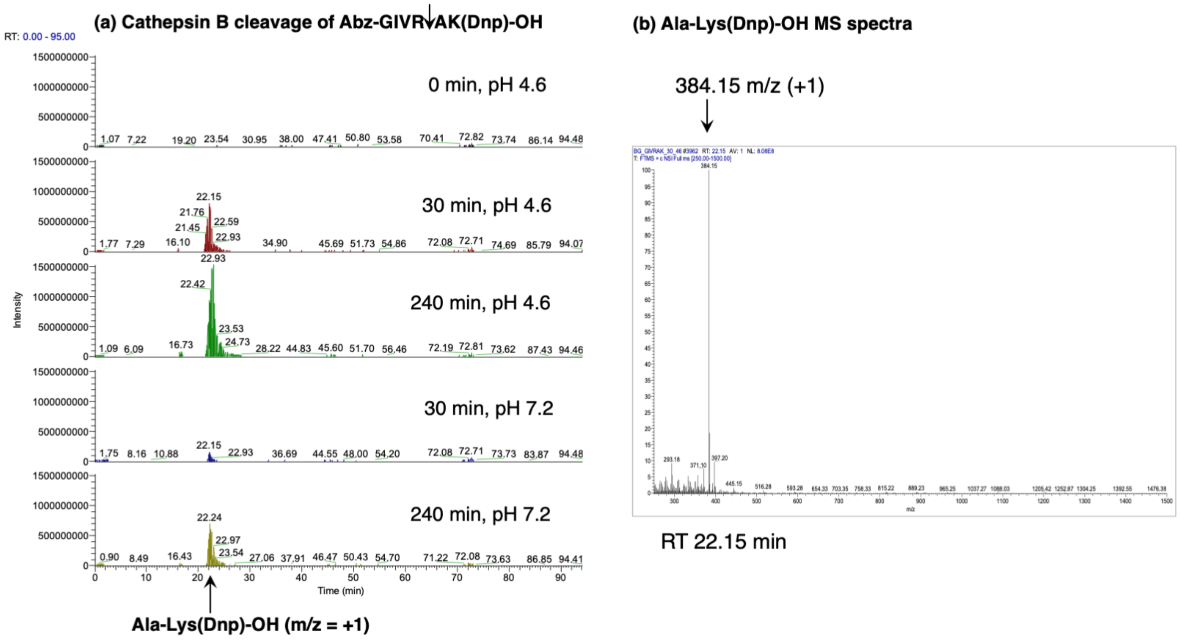

Figure S2. Abz-GIE̲R↓AK(Dnp)-OH cleavage by cathepsin B generates AK(Dnp)-OH illustrated by mass spectrometry. Cathepsin B was incubated with this substrate at pH 4.6 and pH 7.2 at time-course points of 30 min and 240 min, and the TIC illustrates production of the AK(Dnp)-OH cleavage product in panel a. Mass spectra for the DPCP cleavage product of AK(Dnp)-OH m/z (+1) of 384.15 is shown in panel b.

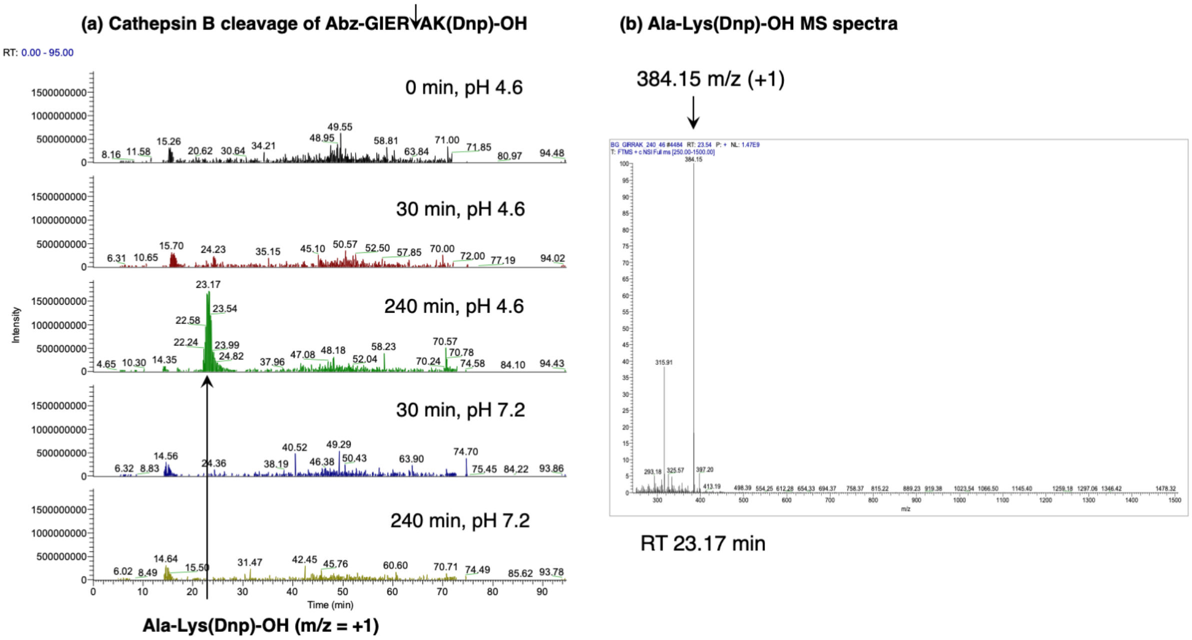

Figure S3. Abz-GIRR↓AK(Dnp)-OH cleavage by cathepsin B generates AK(Dnp)-OH illustrated by mass spectrometry. Cathepsin B was incubated with this substrate at pH 4.6 and pH 7.2 at time-course points of 30 min and 240 min, and mass spectrometry identified the AK(Dnp)-OH cleavage product, shown by its retentaion in the TIC of panel a. Mass spectra for the DPCP cleavage product of AK(Dnp)-OH m/z (+1) of 384.15 is shown in panel b.

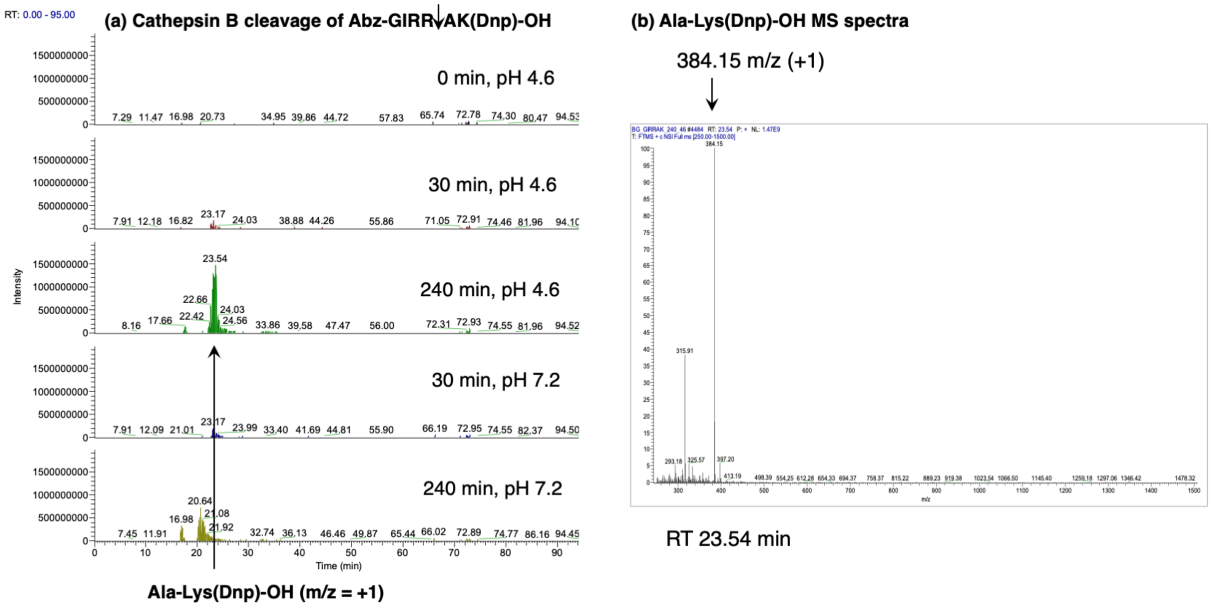

Figure S4. Abz-GnVR↓AK(Dnp)-OH cleavage by cathepsin B generates AK(Dnp)-OH illustrated by mass spectrometry. Cathepsin B was incubated with this substrate at pH 4.6 and pH 7.2 at time-course points of 30 min and 240 min, and mass spectrometry identified the AK(Dnp)-OH cleavage product observed in the TIC (total ion chromatograph) shown in panel a. Mass spectra for the DPCP cleavage product of AK(Dnp)-OH m/z (+1) of 384.15 is shown in panel b.

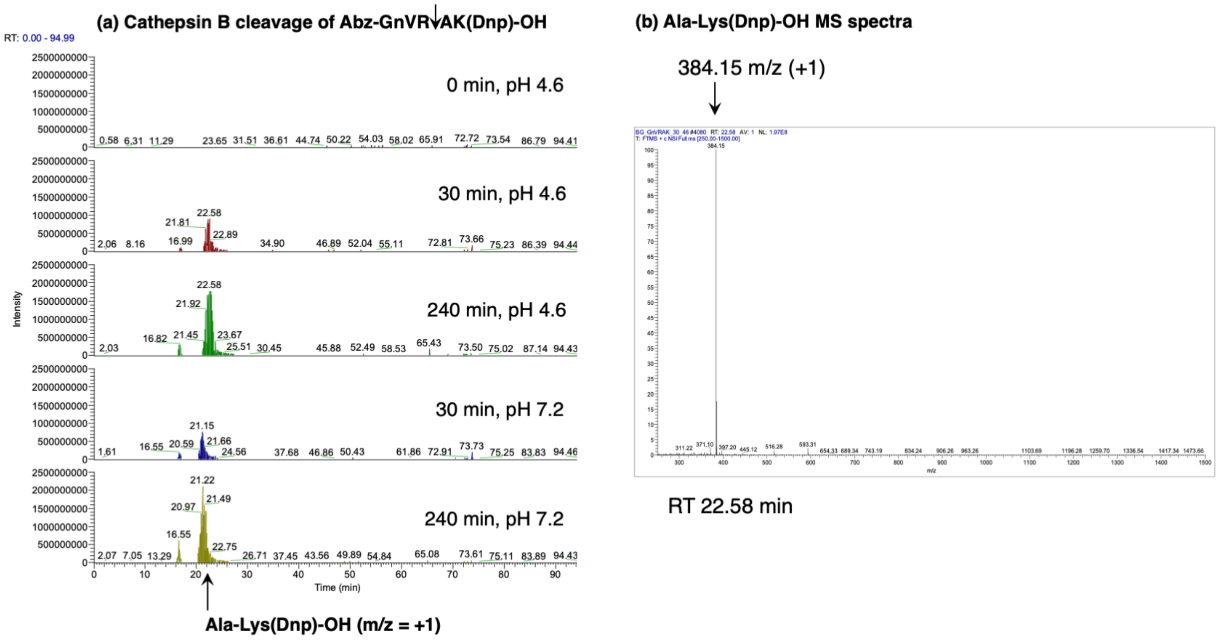

Figure S5. Abz-GIVR↓AK(Dnp)-NH_2 cleavage by cathepsin B generates AK(Dnp)-NH_2 illustrated by mass spectrometry. Cathepsin B was incubated with this substrate at pH 4.6 and pH 7.2 at time-course points of 30 min and 240 min, and TIC shows the retention time (RT) of the AD(Dnp)-NH_2 cleavage product, panel a. Mass spectra for the DPCP cleavage product of AK(Dnp)-NH_2 m/z (+1) of 383.17 is shown in panel b.

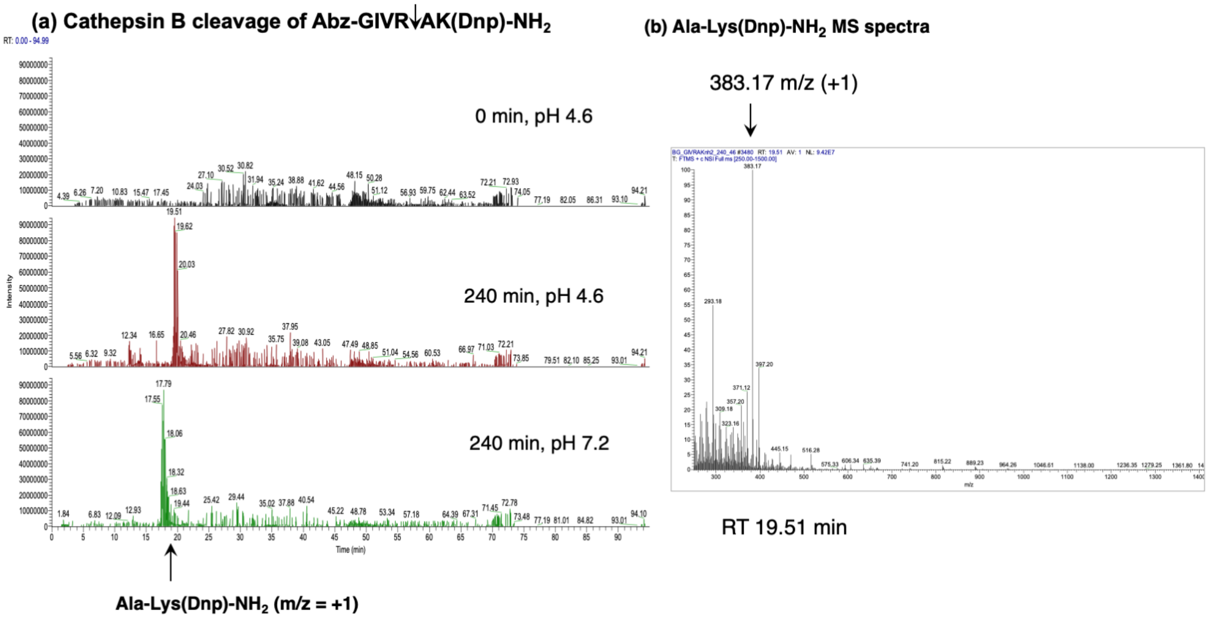

Figure S6. Cathepsin B cleavage of the endopeptidase substrates Abz-GIVRAK(Dnp)-NH_2 and Z-VR-AMC compared to the DPCP substrate Abz-GIVRAK(Dnp)-OH.

<u>(a) pH profile of cathepsin B endopeptidase activity monitored with Abz-GIVRAK(Dnp)-NH_2 and Z-VR-AMC substrates.</u> The full pH profile assessed the pH properties of the DPCP activity of cathepsin B assayed with the Abz-GIVRAK(Dnp)-NH_2 and Z-VR-AMC substrates. Cathepsin B specific activity is illustrated as the percent of its highest activity observed at the pH optimum with each substrate; data are shown as mean $\pm$ SD (n = 3).

<u>(b) pH profile of cathepsin B's Z-VR-AMC endopeptidase activity compared to the enzyme's DPCP activity monitored with Abz-GIVRAK(Dnp)-OH substrate.</u> The pH profile of the Z-VR-AMC endopeptidase activity of cathepsin B and the Abz-GIVRAK(Dnp)-OH DPCP activity were assessed. Cathepsin B specific activity is indicated as percent of its highest activity observed at the pH optimum of the substrate; data are shown as mean $\pm$ SD (n = 3).

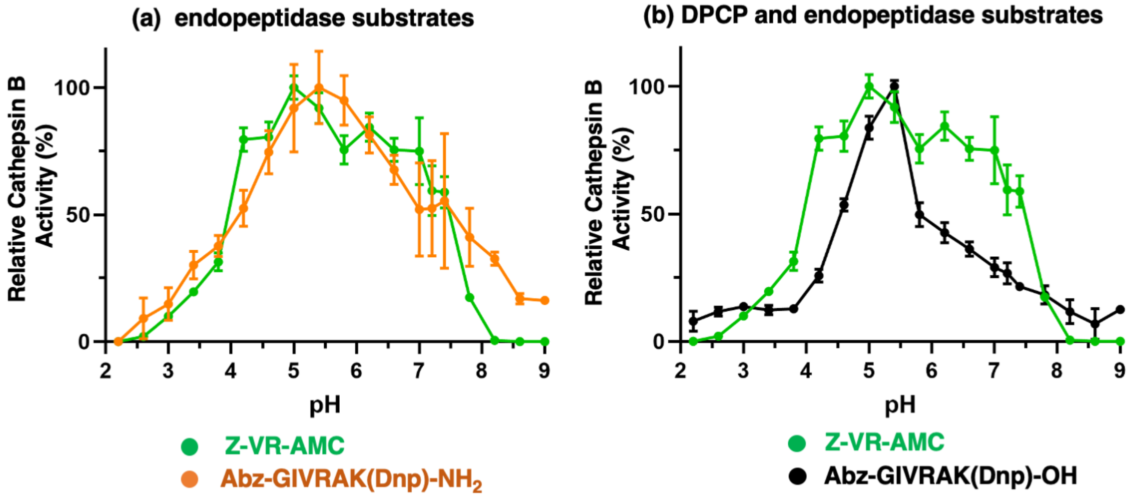

Supplement: Supplementary file 1 — bi2c00358_si_001.pdf [file bi2c00358_si_001.pdf]
